# Ideal Cardiovascular Health in a Nationally Representative Population-Based Sample of Adults in Malawi

**DOI:** 10.5334/gh.986

**Published:** 2021-04-20

**Authors:** Supa Pengpid, Karl Peltzer

**Affiliations:** 1 ASEAN Institute for Health Development, Mahidol University, Salaya, Phutthamonthon, Nakhon Pathom, TH; 2 Department of Research Administration and Development, University of Limpopo, Turfloop, ZA; 3 Department of Psychology, University of the Free State, Bloemfontein, ZA; 4 Department of Psychology, College of Medical and Health Science, Asia University, Taichung, TW

**Keywords:** cardiovascular health, adults, Malawi

## Abstract

**Background:**

Ideal cardiovascular health (ICH) measures four ideal health behaviours (non-smoking, body mass index <25 kg/m^2^, healthy diet, and physical activity) and three health factors (total cholesterol <200 mg/dL, blood pressure <120/<80 mmHg, and fasting blood glucose <100 mg/dL).

**Objective:**

This study aimed to determine the prevalence, distribution, and correlates of ICH among adults in Malawi.

**Methods:**

National cross-sectional survey data of 3,441 persons aged 18–69 years with complete ICH measurements in Malawi in 2017 were analysed.

**Results:**

Almost one in ten (7.4%) of respondents had 0–2 ICH metric), 21.2% 3–4 ICH metrics, and 71.5% 5–7 ICH metrics). Only 3.3% had all seven ICH metrics, 15.3% had intermediate ICH (≥1 metric in the intermediate category and none in the poor category), and 81.5% poor ICH (≥1 metric in poor category). In adjusted logistic regression analysis, older age (50–69 years) (Adjusted Odds Ratio-AOR: 0.25, 95% Confidence Interval-CI: 0.17–0.36) and urban residence (AOR: 0.56, 95% CI: 0.40–0.78) were negatively associated with meeting 5–7 ICH metrics. In addition, in unadjusted analysis, higher education was positively associated with meeting 5–7 ICH metrics.

**Conclusion:**

The proportion of meeting 5–7 ICH metrics was high in Malawian adults. Both high-risk and population-wide intervention programmes targeting older adults and urban residents should be implemented in aiding to improve cardiovascular health in Malawi.

**Highlights:**

- The proportion of meeting 5–7 ideal cardiovascular health (ICH) metrics was 71.5% in Malawian adults (18–69 years, median age 32 years).
- In a sub-group analysis (45–69 years, median age 55 years), 55.9% had 5–7 ideal ICH metrics.
- ICH was lower with increasing age and among urban residents.

## Background

Globally, it is estimated that 31% of all death are attributed to cardiovascular diseases (CVDs), such as heart attacks and stroke, in 2016 [1]. Ischaemic heart disease and stroke were the major causes of disability-adjusted life years (DALYs) in persons 50 years and older in 2019 [2]. More than three-quarters of deaths from CVDs occur in low- and middle-income countries [1].

To reduce CVDs, the American Heart Association (AHA) developed the concept of ideal cardiovascular health (ICH), including seven ideal health behaviours and factors [3,4]. An increasing number of ICH metrics have been found protective against ‘the all-cause and CVD-related mortality risk, incident cardiovascular events, lower prevalence and incidence of non-CVD outcomes such as cancer, depression, and cognitive impairment’ [5]. To our knowledge, there are no national data on ICH in Malawi, a low-income country in Southern Africa. CVDs contribute to 10% of mortality in 2016 in Malawi [6]. In Malawi, a high burden of CVD risk factors has been found, including hypertension and diabetes, smoking, heavy alcohol use, physical inactivity, high salt and sugar intake, low fruit and vegetable intake, and obesity [7,8]. CVD is on the rise in Africa, mainly attributable to an increase in hypertension, smoking, and obesity [9].

Globally, mainly in high-income countries, 32.2% (having 0–2 ideal metrics) had overall poor and 19.6% of participants had 5–7 ICH metrics [10]. Fewer studies have been conducted on ICH in Africa. In rural South Africa (46% HIV positive, 38.7 years mean age), 7% had poor (0–2 metrics),, 40% intermediate (3–4 metrics) and 53% had 5–7 ICH metrics [11], in Uganda (N = 857, ≥16 years, mean age 41.5 years in women and 36.8 years in men), 3.2% had 7 ICH metrics, 50.0% had 5–7 ICH metrics [12], and in urban (n = 596) and rural (n = 326) Ghana (25–70 years, mean age 44 and 45 years in rural and urban women, respectively), urban women 74.7% ≥1 metric in poor category, 25.0% in ≥1 metric in the intermediate category and none in the poor category, 0.3% all 7 ICH metrics [13]. Globally, smoking had the highest prevalence of ICH status (69.1%), followed by fasting blood glucose (FPG) (67.7%), total cholesterol (TC) (51.7%), physical activity (PA) (40.6%), body mass index (BMI) (40.3%), blood pressure (BP) (34.6%), and dietary pattern (12.1%) [10].

Sociodemographic factors associated with ICH may include female sex [63 high income countries-HIC and 28 low- and middle-income countries-LMIC, 10, Brazil, 14, Nepal, 15], younger age [63 HIC and 28 LMIC, 10, Uganda, 12, Brazil, 14, Nepal, 15, China, 16], ethnicity [Brazil, 14], higher education [Uganda, 12, Brazil, 14], higher income [Jamaica, 17, China, 18], lower income [Uganda, 12], rural residence [Ghana, 13, Peru, 19], and geographic region [Brazil, 20]. This study aimed to determine the prevalence, distribution, and correlates of ideal ICH among adults in Malawi in 2017.

## Method

### Sample and procedures

The study design was a national community based cross-sectional survey in Malawi in 2017, using WHO STEPwise approach for assessing risk factors for chronic non-communicable diseases (NCDs), and includes questionnaire data on demographic and behavioural information (Step 1), physical measurements (Step 2) and biochemistry tests (Step 3) [21]. Based on a multistage cluster sample design, the ‘2017 Malawi STEPS Survey’ produced nationally representative population-based data for persons 18–69 years in Malawi [21]. Inclusion criteria was one randomly selected adult household member aged 18–69 per household who was able to provide informed consent. More information on the study methods and data can be publicly accessed [21]. The inclusion criteria for the present study were individuals with no data missing for smoking status, BMI, PA, diet, total TC, FBG, and BP measurements. From 4,187 adults, a total of 3,441 participants with full required information were included. Ethics approval was obtained from the ‘Malawi National Ethics Committee’ and participants provided written informed consent [21].

## Measures

Poor, intermediate and ICH levels for smoking, BMI, PA, diet, TC, BP, and FBG were determined, based on modified AHA definitions [3,4], exact AHA definitions are provided in brackets.

### Cardiovascular health behaviour

*Smoking status*: Smoking is defined as poor if current smoker, intermediate [former ≤12 months] if a past smoker, and ideal if self-report of [never or quit >12 months] never having smoked.

*Body Mass Index* (BMI) (kg/m^2^): BMI is defined poor if ≥30 kg/m^2^, intermediate as 25.0–29.9 kg/m^2^, and ideal BMI is <25 kg/m^2^. Anthropometric measurements were taken by trained healthcare staff using a portable electronic weighing scale and measuring inflexible bars [21].

*Healthy diet*: [poor: 0–1 components, intermediate: 2–3, and ideal: 4–5 components (1: ≥4.5 cups/day fruits and vegetables, 2: ≥3.5 ounce servings/week of fish, 3: <1500 milligrams/day sodium, 4: <450 calories/week sweets/sugar, and 5: ≥3 1-ounce servings/day whole grains)]. Poor healthy diet is defined in this study as <2 servings of fruit and vegetables (FV)/day, intermediate as 2-<4.5 FV/day, and an ideal diet as ≥4.5 FV servings/day.

*Physical activity* (PA): ‘Poor = None, Intermediate = 1–149 min/wk moderate intensity or 1–74 min/wk vigorous intensity or 1–149 min/wk moderate+vigorous, ideal = ≥150 min/wk moderate intensity or ≥75 min/wk vigorous intensity or ≥150 min/wk moderate+vigorous.’ PA was assessed with the Global Physical Activity Questionnaire [22].

### Cardiovascular health factors

*Poor total cholesterol* (TC) is classified as ‘TC ≥6.3 mmol/L (≥240 mg/dL), intermediate is TC 5.2–6.2 mmol/L (200–239 mg/dL) or treated to TC <5.2 mmol/L (<200 mg/dL) and ideal TC is <200 mg/dL and without any cholesterol-lowering medication.’

*Fasting blood glucose (FBG)*: poor FBG is defined as ‘glucose≥ 7.0 mmol/L (≥126 mg/dL), intermediate is glucose 5.6–5.9 mmol/L (100–125 mg/dL) or treated to <100 mg/dL, and ideal is <5.6 mmol/L <100 mg/dL and without any glucose-lowering medication.’

For TC and FBG finger blood samples for biochemistry tests were taken, provided instructions of starving overnight were followed, and TV and FBG were measured using Cardiochek [Report].

*Blood pressure* (BP): poor is defined as ‘BP ≥140/≥90 mmHg, intermediate is systolic BP 120–139 mmHg or diastolic BP 80–89 mmHg or treated to BP <120/<80 mmHg, and ideal BP is defined as BP <120/<80 mmHg and without any antihypertensive medication.’ Of the three BP measurements (taken 3–5 minutes apart) using digital BP machines (Omron M4-I), the last two readings were averaged [21].

The seven ICH items were dichotomised (1 = ideal, 0 = not ideal), and grouped into 0–2, 3–4, and 5–7 ICH metric; 5–7 ICH metric is in the absence of any previous CVD. In addition, three ICH groups were created as follows: ‘ICH is all seven health metrics at ideal levels in the absence of any previous CVD, intermediate ICH is at least one health metric at the intermediate level, but no poor ICH metrics, and poor ICH is at least one of seven ICH metrics at poor level’ [3,4,23]. Ideal health behaviour was defined as the simultaneous presence of 4 ideal health behaviours (adequate PA, nonsmoker, normal BMI, and healthy diet) and ideal health factors as the simultaneous presence of four ideal health factors (non-smokers, normal BP, normal FBG, and normal TC) [3,4,23].

History of CVDs was assessed with the question, ‘Have you ever had a heart attack or chest pain from heart disease (angina) or a stroke (cerebrovascular accident or incident)?’ (Yes, No) [21].

Sociodemographic covariates included age, sex, highest level of formal education, work, and residence status [21].

## Data analysis

Considering the clustered study design, all statistical analyses were conducted with ‘STATA software version 14.0 (Stata Corporation, College Station, TX, USA).’ Only participants with complete ICH assessments were included in the analysis. Chi-square tests were used for estimating differences in proportions and Student’s t-test for differences in means. ICH metrics are described across ideal, intermediate, and poor ICH. Unadjusted and adjusted logistic regressions were used to calculate sociodemographic predictors (age group, sex, educational level, employment status and residence status) of meeting 5–7 ICH metrics, overall and for men and women separately. P-values of below 0.05 were accepted as significant and missing values were excluded from the analysis.

## Results

### Sample characteristics

The sample included 3,441 adults (18–69 years), with a median age of 32 years (interquartile range 24–42), 35.9% were male. More than half of the participants (59.0%) Standard 5 or higher education, 42.9% were employed or students, and 90.0% lived in rural areas. The mean BMI of the respondents was 22.6, the mean systolic BP was 121.1 mmHg, and the prevalence of self-reported CVD was 6.8%. Compared to men, women had lower education, lower employed or student status, lower systolic BP, more likely living in urban areas, more likely having a CVD, more likely taking antihypertensive and lipid-lowering drugs, having a higher BMI, and higher total TC (see Table 1).

**Table 1:** Sample characteristics of participants aged 18-69, Malawi, 2017.

Variable	Total (N = 3441)	Men (n = 1237)	Women (n = 2204)

	%	%	%

All		35.9	64.1
Age (years)			
18–29	44.9	47.5	42.2
30–49	39.8	38.4	41.3
50–69	15.3	14.1	16.6
Education			
Secondary or more	23.5	26.1	20.9*
Standard 5–8	35.5	40.4	30.6
Standard 1–4	31.1	28.1	34.2
None	9.9	5.5	14.3
Employment status			
Nonpaid or unemployed	57.1	71.4	43.2*
Employed or student	42.9	28.6	56.8
Residence			
Rural	90.0	92.0	87.9*
Urban	10.0	8.0	12.1
Self-reported cardiovascular disease	6.8	4.6	9.1*
Use of anti-hypertensive drug	2.4	0.8	4.0*
Use of hypoglycemic drug	0.3	0.4	0.2
Use of lipid-lowering drug	0.1	0.0	0.1*
	**M (SD)**	**M (SD)**	**M (SD)**

Mean systolic blood pressure, mmHg	121.1 (15.9)	122.4 (15.4)*	119.7 (16.2)
Mean body mass index, kg/m^2^	22.6 (4.0)	21.7 (3.2)	23.6 (4.4)*
Mean total cholesterol, mmol/L	3.5 (0.9)	3.3 (0.8)	3.7 (0.9)*
Mean fasting plasma glucose, mmol/L	4.7 (1.0)	4.6 (1.4)	4.8 (1.1)*

* p < 0.05, men versus women.

### Distribution of cardiovascular health metrics

The distribution of the three levels of all seven ICH metrics in the overall adult population and by sex is shown in Table 2. Approximately, 84.0% of Malawian adults reported that they never smoked (70.9% in men and 97.4% in women). More than four in five participants (81.8%) had ideal BMI (90.7% in men and 72.8% in women), and 97.8% had ideal physical activity (99.0% in men and 96.7% in women). A low proportion of healthy diet (≥4.5 servings of fruit and vegetables/day) of 11.3% was reported (9.7% among men and 13.0% among women). Most Malawian adults had ideal total cholesterol (95.0%) and fasting glucose levels (92.7%), while only 46.9% had ideal blood pressure. A significant higher proportion of women had ideal smoking and ideal blood pressure than men, while men had significantly higher ideal BMI, PA and TC than women. Almost one in ten (7.4%) of respondents had poor ICH (0–2 ideal metrics), 21.2% intermediate ICH (3–4 ideal metrics), and 71.5% ideal ICH (5–7 ideal metrics). In a sub-group analysis, persons aged 45–69 years, 55.9% had 5–7 ICH metrics. Only 3.3% had all seven ICH metrics, 15.3% intermediate ICH (≥1 metric in the intermediate category and none in the poor category), and 81.5% had poor ICH (≥1 metric in poor category). Men had better ICH metrics than women in the first ICH measure and women had better ICH than men in the second measure. Compared to the 18- to 44-year-olds, 45- to 69-year-olds had poorer overall ICH metrics as well as six individual ICH, except for fruit and vegetable intake (see Table 2).

**Table 2:** Ideal cardiovascular health (ICH) metrics distribution (prevalence, %).

Health metrics		Total sample (N = 3441)	Men (n = 1237)	Women (n = 2204)	Chi-square *p*-value, Men vs Women	Age: 18–44 years	Age: 45–69 years	Chi-square *p*-value, 18–44 vs 45–69

Smoking	Poor	11.4	20.9	1.7	<0.001	9.9	16.4	<0.001
	Intermediate	4.5	8.1	0.9		2.9	9.4	
	Ideal	84.0	70.9	97.4		87.2	74.2	
Body mass index	Poor	5.0	1.0	9.1	<0.001	4.4	6.7	0.008
	Intermediate	13.2	8.3	18.1		12.3	16.5	
	Ideal	81.8	90.7	72.8		83.3	76.7	
Diet: Fruit and vegetable intake	Poor	71.3	74.8	67.8	0.066	72.2	72.3	0.833
	Intermediate	17.4	15.5	19.3		16.8	17.4	
	Ideal	11.3	9.7	13.0		11.1	10.3	
Physical activity	Poor	1.1	0.5	1.6	0.007	0.7	2.3	<0.001
	Intermediate	1.1	0.5	1.7		0.9	1.7	
	Ideal	97.8	99.0	96.7		98.4	96.1	
Total cholesterol	Poor	0.7	0.2	1.2	<0.001	0.5	1.9	<0.001
	Intermediate	4.2	2.5	6.0		3.2	9.0	
	Ideal	95.0	97.3	92.8		96.3	89.1	
Blood pressure	Poor	15.6	14.9	16.3	0.004	11.0	30.9	<0.001
	Intermediate	37.5	42.1	32.9		38.8	32.1	
	Ideal	46.9	43.0	50.8		50.2	37.1	
Fasting plasma glucose	Poor	1.5	1.4	1.6	0.307	1.3	1.7	<0.001
	Intermediate	5.9	4.7	7.0		4.5	9.3	
	Ideal	92.7	93.9	91.4		94.2	89.0	
0–2 ICH metrics		7.4	4.9	9.9	<0.001	5.9	12.9	<0.001
3–4 ICH metrics		21.2	22.3	20.0		18.4	31.2	
5–7 ICH metrics)		71.5	72.8	70.2		75.7	55.9	
Ideal ICH^a^		3.3	1.6	4.9	<0.001	3.9	1.0	<0.001
Intermediate ICH^b^		15.3	13.5	17.1		16.3	11.4	
Poor ICH^c^		81.5	84.8	78.1		79.8	87.6	

^a^ ‘All seven ICH metrics at ideal levels in the absence of cardiovascular disease (CVD), ^b^ at least one of seven ICH metrics at intermediate levels, no poor ICH metrics in participants without CVD history or if all seven ICH metrics are ideal among persons with a CVD history, ^c^ at least one of seven ICH metrics at a poor level in participants without a CVD history or at least one metric is intermediate or poor among persons with a CVD history’ [10,25].

### Proportion of ideal cardiovascular health metrics

In all, 0.0% had zero, 0.1% one, 0.6% two, 4.5% three, 18.6% four, 40.3% five, 32.3% six and 3.6% all seven ICH metrics (see Table 3). A total of 36.1% participants were ideal on all four health factors, but only 7.3% were ideal on all four health behaviours, the proportion of all four health factors was significantly higher among women (44.2%) than men (28.1%) (p < 0.001) as well as for all four health behaviours (9.0% among women and 5.6% among men) (p < 0.001).

**Table 3:** Distribution of ideal cardiovascular health (ICH) metrics in percent among participants.

Variable	Sample	Proportion of ICH metrics							

	N	0	1	2	3	4	5	6	7

All	3441	0.0	0.1	0.6	4.5	18.6	40.3	32.3	3.6
Age in years									
18–29	1037	0.0	0.0	0.0	2.2	12.9	40.5	40.0	4.4
30–49	1588	0.0	0.1	0.7	4.9	21.0	41.1	28.6	3.5
50–69	816	0.0	0.6	1.9	10.3	28.9	37.3	19.8	1.3
45–69	1108	0.0	0.4	1.9	8.7	26.5	38.4	23.0	1.0
Sex									
Female	2204	0.0	0.1	0.8	4.7	18.3	35.7	35.0	5.3
Male	1237	0.0	0.1	0.4	4.3	18.8	44.8	29.7	1.9
Education									
Secondary or more	861	0.0	0.2	0.7	3.8	17.3	45.1	31.0	1.9
Standard 5–8	1023	0.0	0.0	0.5	3.6	14.7	39.5	37.4	4.3
Standard 1–4	1073	0.0	0.1	0.4	4.7	22.4	38.5	29.9	4.1
None	481	0.0	0.6	1.2	9.2	23.6	37.2	24.8	3.4
Employment status									
Nonpaid or unemployed	1963	0.0	0.2	0.6	4.5	18.1	38.5	33.5	4.6
Employed or student	2204	0.0	0.0	0.6	4.6	19.2	42.6	30.8	2.3
Residence									
Rural	2760	0.0	0.1	0.4	4.3	17.9	40.2	33.5	3.7
Urban	681	0.0	0.3	2.5	6.5	24.9	41.1	21.7	2.9

### Associations with meeting 5–7 ICH metrics

In adjusted logistic regression analysis, older age (50–69 years) (Adjusted Odds Ratio-AOR: 0.25, 95% Confidence Interval-CI: 0.17–0.36) and urban residence (AOR: 0.56, 95% CI: 0.40–0.78) were negatively associated with meeting 5–7 ideal CVH metrics. In addition, in unadjusted analysis, higher education was positively associated with meeting 5–7 ICH metrics (see Table 4).

**Table 4:** Associations with meeting 5-7 ideal cardiovascular health metrics.

Variable	Crude OR (95% CI)	*p*-value	Adjusted OR (95% CI)	*p*-value

All				

Age in years				
18–29	1 (Reference)		1 (Reference)	
30–49	0.48 (0.45, 0.66)	<0.001	0.49 (0.35, 0.68)	<0.001
50–69	0.24 (0.17, 0.33)	<0.001	0.25 (0.17, 0.36)	<0.001
Sex				
Male	1 (Reference)		1 (Reference)	
Female	0.88 (0.72, 1.08)	0.219	0.98 (0.75, 1.27)	0.883
Education				
None	1 (Reference)		1 (Reference)	
Standard 1–4	1.27 (0.67, 2.40	0.459	1.03 (0.69, 1.53)	0.901
Standard 5–8	1.91 (1.03, 3.55)	0.041	1.34 (0.88, 2.04)	0.174
Secondary or more	2.21 (1.20, 4.08)	0.011	1.13 (0.70, 1.84)	0.615
Employment status				
Nonpaid or unemployed	1 (Reference)		1 (Reference)	
Employed or student	1.01 (0.82, 1.24)	0.927	0.97 (0.75, 1.25)	0.808
Residence				
Rural	1 (Reference)		1 (Reference)	
Urban	0.58 (0.43, 0.79)	<0.001	0.56 (0.40, 0.78)	<0.001
**Men**				

Age in years				
18–29	1 (Reference)		1 (Reference)	
30–49	0.48 (0.29, 0.79)	0.004	0.49 (0.29, 0.82)	<0.001
50–69	0.19 (0.11, 0.32)	<0.001	0.20 (0.11, 0.36)	<0.001
Education				
None	1 (Reference)		1 (Reference)	
Standard 1–4	1.38 (0.96, 1.98)	0.085	0.83 (0.40, 1.69)	0.599
Standard 5–8	1.91 (1.32, 2.77)	<0.001	1.20 (0.60, 2.43)	0.605
Secondary or more	1.64 (1.11, 2.43)	0.013	1.23 (0.60, 2.50)	0.571
Employment status				
Nonpaid or unemployed	1 (Reference)		1 (Reference)	
Employed or student	1.11 (0.76, 1.62)	0.577	1.03 (0.67, 1.57)	0.904
Residence				
Rural	1 (Reference)		1 (Reference)	
Urban	1.10 (0.72, 1.66)	0.666	1.04 (0.67, 1.62)	0.855
**Women**				

Age in years				
18–29	1 (Reference)		1 (Reference)	
30–49	0.49 (0.36, 0.66)	<0.001	0.48 (0.35, 0.67)	<0.001
50–69	0.30 (0.21, 0.43)	<0.001	0.29 (0.19, 0.43)	<0.001
Education				
None	1 (Reference)		1 (Reference)	
Standard 1–4	1.49 (0.98, 2.27)	0.063	1.19 (0.75, 1.89)	0.464
Standard 5–8	1.96 (1.24, 3.10)	0.004	1.50 (0.89, 2.51)	0.125
Secondary or more	1.20 (0.69, 2.08)	0.522	0.94 (0.51, 1.74)	0.839
Employment status				
Nonpaid or unemployed	1 (Reference)		1 (Reference)	
Employed or student	0.83 (0.65, 1.07)	0.147	0.90 (0.68, 1.19)	0.456
Residence				
Rural	1 (Reference)		1 (Reference)	
Urban	0.40 (0.28, 0.56)	<0.001	0.40 (0.27, 0.59)	<0.001

OR = Odds Ratio; CI = Confidence Intervals.

## Discussion

To our knowledge, this is the first national study on the prevalence and distribution of ICH metrics in Malawi. In this nationally representative sample in Malawi, the prevalence of poor ICH (0–2 ideal metrics) (7.4%), and 5–7 ICH metrics (71.5%) in the total adult population (18–69 years, median age 32 years) and poor ICH (12.9%) and 5–7 ICH metrics (55.9%) in a subsample (45–69 years, median age 55 years), was better than globally, mainly in high-income countries (0–2 ICH 32.2% and 5–7 ICH metrics 19.6%) [8]. It was also better than in rural South Africa (46% HIV positive, 38.7 years mean age) (7% had poor, 0–2 ideal metrics), and 53% 5–7 ICH metrics [11], and in Uganda (≥16 years, mean age 41.5 years in women and 36.8 years in men, 50.0% had 5–7 ICH metrics) [12]. The proportion of all seven ICH metrics (3.3%), intermediate ICH (≥1 metric in the intermediate category and none in the poor category) (15.3%) and poor ICH (≥1 metric in poor category) (81.5%) in this study, was similar to a study in Uganda (N = 857, ≥16 years, mean age 41.5 years in women and 36.8 years in men), (3.2% had 7 ICH metrics) [12], urban women in Ghana (mean age 45 years) 0.3% all seven ICH metrics, 25.0% in ≥1 metric in the intermediate category and none in the poor category 74.7% ≥1 metric in poor category [13], and in rural area in Northwest China all 7 ICH metrics (0.0%), intermediate (no poor health metrics and at least one intermediate) (18.0%), poor (any poor ICH metric) (82%) [16]. Our findings indicate that the prevalence of ICH is high but still efforts are needed to promote ICH to prevent CVD in Malawi.

Similar to the four best global estimates [10], this study showed that PA (97.8%), TC (95.0%), smoking (84.0%) and FBG (92.7%) had the highest prevalence of ideal status, while similar to the poorest global estimates [10], healthy diet (11.3%) had the poorest prevalence of ideal status in this study. The estimates of ideal PA (97.8%) in this study seem higher than global estimates of PA (40.6%) and ideal BMI (81.8%) are double of the global figures of ideal BMI (40.3%) [10]. In the 2009 STEPS national survey (24–64 years) in Malawi, a similar rate of ideal BMI (78.1%) was observed [24]. The high prevalence of ideal PA (97.8%) in this national study seems to be confirmed in the 2009 STEPS survey in Malawi (91.5% physically active) [25]. A low ideal healthy diet (fruit and vegetable consumption) (11.3% <4.5 servings/day) was also found in the 2009 Malawi STEPS survey (2.5% <5 servings/day) [26]. The proportion of poor smoking was 20.9% among men and 1.7% among women in this study, which is lower than the 2009 Malawi STEPS survey (25.9% among men and 2.9% among women) [25]. Poor BP (15.6%) was in this study (18–69 years) lower than in the 2009 Malawi STEPS survey (33.2%, hypertension, 25–64 years) [27]. Poor and intermediate FBG (7.4%) and poor and intermediate TC (4.9%) were similar to the 2009 survey (impaired and raised FBG 9.8% and 8.7% TC ≥5.0 mmol/L) [25,28]. Similar to the 2009 STEPS survey, smoking and raised blood pressure were more frequent in men than in women, while obesity and raised TC occurred more often in women than in men [25].

Similar to a study in Northwest China [16], this study found that the proportion of having all four ideal health factors (36.1%) was significantly higher than those with all four ideal health behaviours (7.3%). Both, the proportion of ICH health factors and ICH health behaviours were higher in women than in men, while in a study in rural Uganda, ICH health factors were higher in men than in women and ICH health behaviours were higher in women than in men [12]. This result may pinpoint that the promotion of healthy behaviours should be emphasised to improve ICH [16]. A healthy diet (fruit and vegetable intake) was the least prevalent health metric (11.3%) in this study. Lack of affordability and availability of fruit and vegetables may be an influencing factor for the low intake of FV [11,29]. Consequently, population strategies and interventions targeting persons with low fruit and vegetable consumption are urgently needed to improve ICH in Malawi.

Consistent with previous research [10,12,13,14,15,16,19], ICH was higher among younger age groups, rural residence, and those with higher education in unadjusted analysis.

The nonsignificant sex differences may be explained by on the one hand, women had a higher rate of ideal smoking and ideal BP than men, and on the other hand, men had a higher rate of ideal BMI, PA and TC than women.

To improve ICH in the Malawian, ICH behaviours should be improved through multidisciplinary interventions in living individuals, health educators, policy makers, and public health professionals [30]. Comprehensive interventions may target promotion of healthy diets, body weight control, smoking cessation, and screening and control of high levels of blood pressure and blood sugar [31]. Findings of the study may help in the NCD policy and plan of action in Malawi.

Most cases of hypertension and diabetes remain undiagnosed, untreated, or inadequately controlled. Our review demonstrated that there is an increased risk of hypertension and diabetes at a younger age and often in individuals with relatively low or normal BMI.

Given limited human and financial resources, innovative models of care are required to mitigate the growing burden of NCDs in Malawi. More broadly, we identified two innovative models of care integration across Malawi [20,21]. The first model of care in southern Malawi is an integrated chronic care clinic that utilizes an HIV program as a platform for various chronic care screening and treatment (hypertension, diabetes, asthma, and epilepsy, regardless of HIV status) [21]. This model of care allows patients with chronic conditions or HIV to be screened and treated at a single facility during a single visit. The second model of care from central Malawi reported leveraging an HIV service platform for the screening and treatment of hypertension [20]. These studies documented that the integration of hypertension and diabetes screening into an HIV clinic is feasible despite various challenges including frequent stock out and dispensing of NCD drugs, patient flow, workload, and issues related to data monitoring and evaluation [20,21].

A recent NCD model of care review in sub-Saharan Africa also revealed that leveraging existing human resources, decentralization of NCD care to primary health facilities, task redistribution including to lay health cadres, designing patient-centered quality of care, and continued training and mentorship are key to successes of NCD treatment and control [24]. Although community screening and sensitization on the need for NCD care is critical, strategies are needed to ensure better linkage and retention into care [17].

The study strength is the large nationally representative sample and using standardized WHO STEPS methodology and measures. Study limitations include the cross-sectional design, the assessment of some variables was assessed by self-report. Furthermore, we included only one healthy diet component (fruit and vegetable consumption) and not all five components of AHA healthy diet. The participants included two-thirds women, but data were weighted for sex and age to the Malawian population.

## Conclusion

The proportion of 5–7 ICH metrics was high in Malawian adults. Both high-risk and population-wide intervention programmes targeting older adults and urban residents should be implemented in aiding to improve ICH in Malawi.

## Data Accessibility Statement

‘The data for the current study are publicly available at the World Health Organization NCD Microdata Repository (URL: https://extranet.who.int/ncdsmicrodata/index.php/catalog).’

## Abbreviations

AHA = American Heart Association; BP = Blood pressure; BMI = Body Mass Index; CVD = Cardiovascular disease; DALYs = Disability adjusted life years; FBG = Fasting blood glucose; FV = Fruit and Vegetables; ICH = Ideal Cardiovascular Health; NCD = non-communicable disease; PA = Physical activity; STEPS = *STEPwise* approach to *Surveillance*; TC = *Total cholesterol*.
